# A putative 2,3-bisphosphoglycerate-dependent phosphoglycerate mutase is involved in the virulence, carbohydrate metabolism, biofilm formation, twitching halo, and osmotic tolerance in *Acidovorax citrulli*

**DOI:** 10.3389/fpls.2022.1039420

**Published:** 2022-11-09

**Authors:** Jongchan Lee, Jeongwook Lee, Yongmin Cho, Junhyeok Choi, Sang-Wook Han

**Affiliations:** Department of Plant Science and Technology, Chung-Ang University, Anseong, South Korea

**Keywords:** *acidovorax citrulli*, bacterial fruit blotch, proteomics, 3-bisphosphoglycerate-dependent phosphoglycerate mutase, virulence

## Abstract

*Acidovorax citrulli* (*Ac*) is a gram-negative bacterium that causes bacterial fruit blotch (BFB) disease in cucurbit crops including watermelon. However, despite the great economic losses caused by this disease worldwide, *Ac*-resistant watermelon cultivars have not been developed. Therefore, characterizing the virulence factors/mechanisms of *Ac* would enable the development of effective control strategies against BFB disease. The 2,3-bisphosphoglycerate-dependent phosphoglycerate mutase (BdpM) is known to participate in the glycolysis and gluconeogenesis pathways. However, the roles of the protein have not been characterized in *Ac*. To elucidate the functions of BdpmAc (Bdpm in *Ac*), comparative proteomic analysis and diverse phenotypic assays were conducted using a *bdpmAc* knockout mutant (*bdpmAc:Tn*) and a wild-type strain. The virulence of the mutant to watermelon was remarkably reduced in both germinated seed inoculation and leaf infiltration assays. Moreover, the mutant could not grow with fructose or pyruvate as a sole carbon source. However, the growth of the mutant was restored to levels similar to those of the wild-type strain in the presence of both fructose and pyruvate. Comparative proteomic analyses revealed that diverse proteins involved in motility and wall/membrane/envelop biogenesis were differentially abundant. Furthermore, the mutant exhibited decreased biofilm formation and twitching halo size. Interestingly, the mutant exhibited a higher tolerance against osmotic stress. Overall, our findings suggest that BdpmAc affects the virulence, glycolysis/gluconeogenesis, biofilm formation, twitching halo size, and osmotic tolerance of *Ac*, suggesting that this protein has pleiotropic properties. Collectively, our findings provide fundamental insights into the functions of a previously uncharacterized phosphoglycerate mutase in *Ac*.

## Introduction

*Acidovorax citrulli* (*Ac*) is a gram-negative, rod-shaped, and seed-borne bacterium belonging to the beta subdivision of the Proteobacteria (Schaad et al., 1978; Silva et al., 2016). *Ac* is the causative agent of bacterial fruit blotch (BFB) disease, which affects cucurbit crops worldwide. This disease can occur at any stage of watermelon growth and the symptoms can be detected in various parts of the plant (Rahimi-Midani and Choi, 2020). When a watermelon seedling is infected by *Ac*, the cotyledon and leaf develop a water-soaked lesion, and the seedling wilts and dies after a few days (Burdman et al., 2005). In infected fruit, the surface of the fruit develops a water-soaked area and later becomes blackened, which is accompanied by the decay of the inner flesh of the fruit. *Ac* strains can be divided into two groups: Group I is mainly isolated from non-watermelon cucurbits such as melon, and Group II is mainly isolated from watermelon (Zivanovic and Walcott, 2017). BFB greatly impacts fruit production and poses a major threat to the cucurbit industry worldwide (Shrestha et al., 2013; Islam et al., 2020). Nevertheless, there are currently no effective methods to control this disease because resistant cultivars/lines have not been identified. Therefore, characterizing the virulence factors and mechanisms of *Ac* infection is crucial for the development of effective disease control strategies.

Diverse virulence factors of *Ac* have already been reported in previous studies. Many gram-negative phytopathogens use the type III secretion system (T3SS) to directly secrete effector proteins into the host cell to promote their growth or suppress host defenses (Alfano and Collmer, 2004). In *Ac, hrpX* and *hrpG*, which play key roles in the regulation of the T3SS, are essential virulence factors (Zhang et al., 2018). The type II secretion system, which acts as an enzyme or toxin secretory pathway into the extremal environment (Douzi et al., 2012), is also crucial for virulence in *Ac* (Zivanovic and Walcott, 2017). Gram-negative bacteria also possess type IV pili, which are flexible filaments that enable bacterial adhesion to various surfaces (Craig et al., 2004). *Ac* cells with mutations in type IV pili-related genes exhibited reductions in twitching motility and virulence (Rosenberg et al., 2018). Flagella are also known to contribute to the virulence of Group I strains of *Ac* (Bahar et al., 2011). Additionally, quorum sensing, a cell-to-cell communication system in bacteria that can regulate gene expression, is also a well-known virulence mechanism in gram-negative pathogenic bacteria including *Ac* (De Kievit and Iglewski, 2000; Wang et al., 2016). Previous studies have also reported that a ferric uptake regulator, *yggS* family pyridoxal 5’-phosphate binding protein, a bifunctional chorismate mutase/prephenate dehydratase, and a pyridoxal phosphate-dependent aminotransferase contribute to virulence in *Ac* (Liu et al., 2019; Kim et al., 2020; Lee et al., 2021; Wang et al., 2022). Although several studies have characterized *Ac* virulence, additional efforts are still needed, as many virulence-related genes in *Ac* have not been studied yet.

The glycolysis pathway is a catabolic process in which 6-carbon glucose molecules are converted to 2-carbon pyruvate molecules, resulting in the release of ATP (Ganapathy-Kanniappan and Geschwind, 2013). The generated pyruvate can then enter the tricarboxylic acid (TCA) cycle to generate more ATP (Rui, 2014). In contrast, the gluconeogenesis pathway is an anabolic process in which 2-carbon pyruvate molecules are converted to 6-carbon glucose molecules (Mapes and Harris, 1976). Phosphoglycerate mutases (PGMs) are critical enzymes in both glycolysis and gluconeogenesis. This enzyme catalyzes the interconversion of 3-phosphoglycerate and 2-phosphoglycerate in both the glycolysis and gluconeogenesis pathways (Davies et al., 2011). Therefore, PGMs are responsible for synthesizing both glucose and pyruvate in the glycolysis and gluconeogenesis pathways. The PGMs can be categorized into two groups without sequence similarities: cofactor-dependent phosphoglycerate mutases (dPGMs) and cofactor-independent phosphoglycerate mutases (iPGMs) (Zhang et al., 2004). dPGMs are found in all vertebrates, some invertebrates, fungi, and some gram-negative bacteria. iPGMs are found in all plants, algae, some invertebrates, fungi, and some gram-positive bacteria (Jedrzejas, 2000). However, dPGMs are needed for the activation of the 2,3-bisphosphoglycerate, whereas iPGMs are not (Fraser et al., 1999). Mice infected with a mutant strain of *Staphylococcus aureus* lacking iPGM exhibited milder disease symptoms (Radin et al., 2019). Moreover, the dPGM of *Burkholderia phymatum* plays a crucial role in the formation of root nodules in *Mimosa pudica* (Chen et al., 2012). However, the functions of PGMs in *Ac* are yet to be elucidated.

The present study characterized the functions of a putative 2,3-bisphosphoglycerate-dependent phosphoglycerate mutase in *Ac* (BdpmAc) belonging to the dPGM group in the KACC17005 strain, which has an annotated genome (Park et al., 2017). To examine the functions of BdpmAc in *Ac*, pathogenicity and growth assays in M9 minimal media with a sole carbon source were conducted. Additionally, label-free shotgun comparative proteomic analysis was conducted to explore the potential cellular mechanisms related to BdpmAc. Based on our proteomic analysis data, biofilm formation, twitching halo size, and osmotic stress assays were investigated. Through these phenotype assays, our findings demonstrated that BdpmAc is involved in virulence and other key phenotypes in *Ac.*

## Materials and methods

### Bacterial strains and growth conditions

*Ac* strain KACC17005, which belongs to Group II, was used as the wild-type strain in this study. *Ac* was grown in Tryptic Soy Broth (30 g/L) (TSB) or TSB (30 g/L) supplemented with 1.5% Agar (TSA) at 28°C. The *E. coli* strains EC100D and DH5 were used to identify the Tn5 transposon insertion site and for cloning, respectively. *E. coli* strains were grown in Luria-Bertani broth (LB) (Tryptone 10 g/L, NaCl 10 g/L, Yeast extract 5 g/L) or LB supplemented with 1.5% agar at 37°C. In this study, M9 media was used as a minimal medium. For the selection of bacteria harboring genes of interest, appropriate antibiotics were added to each medium at the following final concentrations: rifampicin, 50 μg/mL; ampicillin, 100 μg/mL; kanamycin, 50 μg/mL; gentamicin, 10 μg/mL.

### Selection of *bdpmAc:Tn via* Tn mutant library screening and generation of BdpmAc complemented strain

The bacterial strains and plasmids used in this study are summarized in **Supplementary Table 1**. An *Ac* mutant with decreased virulence was selected by screening the EZ-Tn™ insertional library as previously reported (Kim et al., 2020). After selecting the mutant, the Tn insertion site was identified by following the manufacturer’s instructions (Lucigen, Middleton, WI, USA). A putative 2,3-bisphosphoglycerate-dependent phosphoglycerate mutase (Accession No. ATG93870) was disrupted by Tn and the identified mutant was named *bdpmAc:Tn.* To generate a complemented strain, the full-length *bdpmAc* gene from the wild-type strain was amplified with *bdpmAc* **-**specific primers (5’-ggtaccatgcacaaactcgtcctgat-3’ and 5’-cgtcccagggcaaggcccaccaccaccaccaccactgaagctt-3’) and cloned into the pGem-T easy vector (Promega, Madison, Wisconsin, USA) to obtain pGem-*bdpmAc*. The cloned gene was confirmed by Sanger sequencing. Subsequently, the confirmed gene was cloned again into the pBBR1-MCS5 plasmid (Kovach et al., 1995), which contains the *LacZ* promoter for expression of cloned genes, thus obtaining pMCS5-*bdpmAc*. Finally, pMCS5-*bdpmAc* was introduced into *bdpmAc:Tn*. This insertion was confirmed by polymerase chain reaction (PCR) with *bdpmAc* **-**specific primers. The successful insertion of pMCS5-*bdpmAc* resulted in the *bdpmAc:Tn*(BdpmAc) complemented strain. To avoid the side effects from the vector, pBBR1-MCS5 was introduced into *Ac* and *bdpmAc:Tn* to generate *Ac*(EV) and *bdpmAc:Tn* (EV), respectively. The generated strains were confirmed by PCR with pBBR1-MCS5 specific primers.

### Virulence assay

*Citrullus lanatus* var. *vulgaris* line SBA seeds, which were kindly provided by Partner Seed Company (Gimje, Korea), were used for the virulence assay. Two inoculation assays were performed in this study: germinated-seed inoculation and leaf infiltration. The germinated seed inoculation assay was conducted as previously reported (Kim et al., 2020). Briefly, the watermelon seeds were germinated on water-soaked filter paper for two days. The *Ac* strains were cultured on TSA at 28°C, harvested using cotton swabs, and suspended in 10 mM MgCl_2_. The bacterial suspension was adjusted to an OD_600nm_ of 0.3 (approximately 10^8^ colony forming units (CFUs)/mL), and serially diluted (10^-2^) with the same buffer to reach a 10^6^ CFU/mL concentration. The germinated watermelon seeds were soaked with the prepared bacterial suspension and incubated with gentle agitation for 1 hour at 22°C. The inoculated seeds were sown in 50 pot trays with autoclaved soil and grown for 7 days. The disease severity of inoculated watermelon seedlings was measured every day for 7 days. The disease severity was evaluated based on a 0–2 scale, where 0 indicates no symptoms, 1 indicates the occurrence of spots, and 2 indicates wilting. The disease index was calculated as follows: [(number of plants with no symptoms)×0 + (number of plants with spots)×1 + (number of plants presenting wilting)×2]/Total number of plants. Five independent experiments with ten biological replicates were conducted per strain. The leaf infiltration experiments were conducted as previously reported (Lee et al., 2021). Briefly, the watermelon seeds were grown in a growth chamber until they reached the four true leaves stage. A bacterial suspension was then prepared with 10^5^ CFU/mL (final concentration) in 10 mM MgCl_2_. The prepared inoculant was directly infiltrated into the first and second true leaves of the watermelon seedlings using a needleless syringe. To monitor the bacterial growth, the infiltrated leaves were punched using 0.4 cm diameter cork borers, after which two leaf disks were ground in 200 µL of 10 mM MgCl_2_ using tissue grinders to extract the bacterial cells. The extracted bacterial cells were serially diluted, dotted onto TSA containing the proper antibiotics, and incubated at 28°C for two days. Five independent experiments were conducted with three biological replicates per strain.

### Growth assay

The growth of *Ac* strains was measured in TSB and M9 containing diverse carbon sources, and appropriate antibiotics were added to the media. For TSB, the *Ac* strains were grown on TSA (1.5% agar). The grown bacterial cells were harvested using cotton swabs and washed twice with distilled water. After washing, each strain was resuspended and adjusted to an OD_600nm_ of 0.3 (approximately 10^8^ CFU/mL). The adjusted bacterial cells were diluted (10^-3^) with TSB to achieve a 10^5^ CFU/mL concentration and incubated in a shaking incubator at 28°C. Bacterial growth was monitored every 12 hours for five days using a spectrophotometer. For minimal media, *Ac*(EV), *bdpmAc:Tn*(EV), and *bdpmAc:Tn*(BdpmAc) were grown in six different conditions with appropriate antibiotics (M9 with glucose, M9 with fructose, M9 with pyruvate, M9 with fructose and pyruvate, M9 with mannitol, and M9 with sucrose). The final concentration of each supplemented carbon source was 0.4% in the medium. In M9 with fructose and pyruvate, each carbon source was added at a 0.2% concentration to adjust the entire carbon source to 0.4%. The bacterial cells were washed twice and adjusted to an OD_600nm_ of 0.05 in the minimal media and cell growth was monitored for ten days at 24 h intervals. For this assay, three biological replicates were examined for each strain, and at least four independent experiments were conducted.

### Comparative proteomics

Proteomic analysis was performed at the BT Research Facility Center, Chung-Ang University. The *Ac* and *bdpmAc:Tn* strains were used for label-free shotgun comparative proteomic analysis. Sample preparation, protein extraction, and peptide generation/quantification were performed as reported previously (Kim et al., 2020). Concretely, the three biological replicates from each sample (a total of six samples) were used for the analysis. Bacterial cells were grown in TSB, collected at an OD_600nm_ of 0.6 (approximately 2×10^8^ CFU/mL) using the centrifuge (7,300 g, 15 minutes, 28 °C), and washed twice with 50 mM Tris-HCl (pH 7.8). The washed cells were suspended in lysis buffer (6 M guanidine HCl, 10 mM dithiothreitol, 50 mM Tris-HCl pH 7.8) and sonicated with an ultrasonic processor (Colo Parmer, Vernon Hills, IL, USA). After sonication, the total soluble proteins were precipitated using trichloroacetic acid and treated with trypsin to obtain tryptic digested peptides. The peptides were cleaned using a Sep-Pak Vac 1cc tC18 cartridge (Waters, Milford, MA, USA) and quantified using the BCA assay kit (Thermo Fisher Scientific, Rockford, IL, USA). Next, 1 μg of the samples was analyzed using a split-free nano LC system (EASY-nLC II; Thermo Fisher Scientific, Bremen, Germany) linked to an LTQ Velos Pro instrument (Thermo Fisher Scientific) under nanospray ion mode. To separate the tryptic digested peptides, the samples were loaded onto a 7.5 cm column packed with MAGIC C18AQ 200A (Michrom BioResources, Auburn, CA, USA) and eluted for 420 minutes (300 nL/min flow rate) under the following water/acetonitrile gradient conditions: buffer A, 100% water with 0.1% formic acid; buffer B, 100% acetonitrile with 0.1% formic acid; 7% of buffer B for 5 minutes, 35% of buffer B for 380 minutes, 80% of buffer B for 10 min, and 7% of buffer B for 25 minutes. To obtain the full mass spectra, six data-dependent ms/ms scans were employed with an m/z 300–2000 mass range. The ion charge state selection was allowed for 2^+^ and 3^+^. Dynamic exclusion was permitted under the following conditions: repeat count, 1; repeat duration, 0.5 minutes; elimination duration, 3 minutes. The maximum value of the six most intense ions from each full mass scan was sequentially selected for fragmentation. The mass spectrometry data have been deposited to the ProteomeXchange Consortium via the PRIDE (Perez- Riverol et al., 2022) partner repository with the dataset identifier PXD035156.

Proteins were identified and quantified according to a previously established protocol (Kim et al., 2020). The Thermo Proteome Discoverer software (ver. 1.3.0.399) coupled with the SEQUEST search algorithm was used to identify proteins based on *Ac* strain KACC17005 genome data (Accession No. CP023687) obtained from the NCBI database. The target-decoy strategy was used in this analysis to improve reliability (Elias and Gygi, 2007). Two missed cleavages were allowed and the results were deemed significant at a 0.01 false discovery rate. For mass accuracy, the precursor was 100 ppm and the probability score was more than 20. After identifying the proteins, the Scaffold 4 software (Proteome Software, Portland, OR, USA) was used to conduct comparative analyses. The peptide spectrum matches (PSMs) were used for comparison (Choi et al., 2008). PSMs from each protein were normalized to the total PSMs from each biological replicate. The average value of the PSMs from the three biological replicates was used to identify differentially abundant (more than 2-fold change) proteins between *Ac* and *bdpmAc:Tn*. Pairwise comparisons were conducted *via* Student’s t-test (P<0.05). Clusters of orthologous groups (COG) analysis was used for classifying the differentially abundant proteins (Tatusov et al., 2000).

### Osmotic stress assay

For the osmotic stress assay, the *Ac* strains were exposed to 2.5% NaCl and 40% sorbitol. Both assays were conducted as previously described (Kim et al., 2020; Park et al., 2021). In the NaCl treatment, the bacterial suspension was adjusted to an OD_600nm_ of 0.1 (approximately 3 × 10^7^ CFU/mL) and exposed to 2.5% NaCl for 20 minutes and 40% sorbitol for 40 minutes in TSB. Then, the exposed cells were serially diluted, dotted on TSA (1.5% agar) with appropriate antibiotics, and incubated at 28°C for 2 days, after which the bacterial colonies were counted. TSB alone was used as a negative control. To calculate survivability, we obtained the ratio of the viable cells from the treatments to those from the control. Three biological replicates were analyzed for each strain and seven and five independent experiments were conducted for NaCl and sorbitol, respectively.

### Biofilm formation

Biofilm formation capacity was measured as described in a previous study (Kim et al., 2020) *Ac* strains were suspended in TSB, adjusted to an OD_600nm_ of 0.3 (10^8^ CFU/mL), and diluted (10^-3^) (10^5^ CFU/mL). The prepared suspension was cultured in a 96-well polyvinyl chloride plate for one and three days at 28°C without agitation. After incubation, the cell culture supernatants were removed and washed one time using autoclaved water. Crystal violet (0.1%) was used to stain the remaining cells for 30 minutes. After washing the cells two times with autoclaved water, the stained cells were resuspended in 95% ethanol with gentle shaking. After 20 minutes, the resuspended cells were measured using a Spectramax 190 microplate reader (Molecular Devices, Sunnyvale, CA, USA) at 590 nm. A total of 20 biological replicates of each strain were used in this assay and eight independent experiments were conducted.

### Twitching halo

The twitching halo production assay was conducted as described in a previous study (Kim et al., 2020). *Ac* strains were cultured in TSA, harvested, and adjusted to an OD_600nm_ of 0.3 (approximately 10^8^ CFU/mL). Next, 5 μL of bacterial suspension was dotted on TSA (0.5% agar) and the samples were incubated at 28°C. After two days, the diameter of the colonies and the diameter of the twitching halo were measured under a LEICA M205 C microscope (LEICA, Wetzlar, Germany). Three biological replicates of each strain were employed and eight independent experiments were conducted.

### Statistical analysis

Pairwise comparisons were conducted *via* Student’s *t*-test or one-way analysis of variance (ANOVA) with Tukey’s HSD (honestly significant difference) test using the SPSS 12.0K software (SPSS, Inc., Chicago, IL, USA). A *p*-value < 0.05 was considered statistically significant.

## Results

### BdpmAc is an essential virulence factor of *Ac*

**Figure 1:**
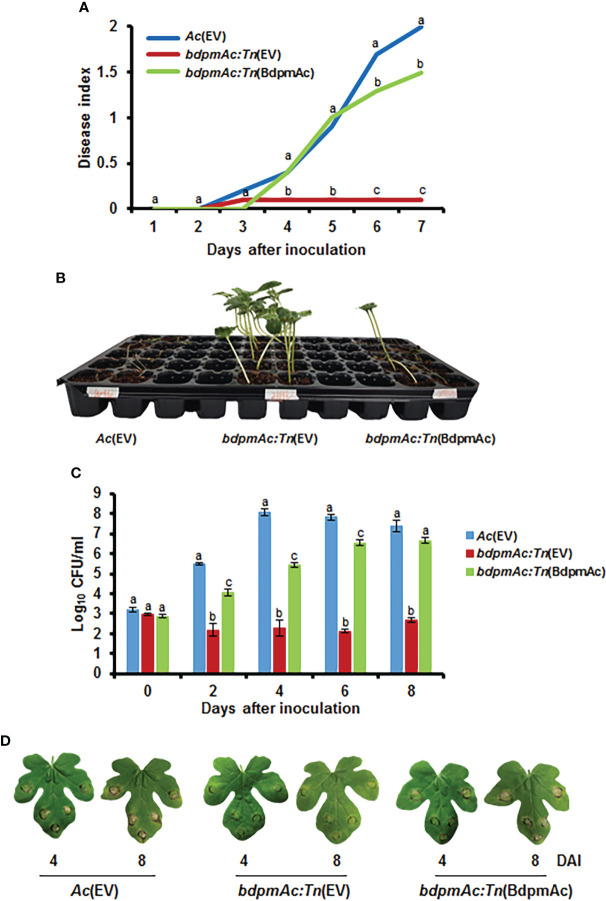
Virulence assay for *Ac*(EV), *bdpmAc:Tn*(EV), and *bdpmAc:Tn*(BdpmAc). **(A)** Disease index of the inoculated germinated seeds at 7 days after inoculation. Disease index: [(number of plants with no symptoms)×0 + (number of plants with spots)×1 + (number of plants presenting wilting)×2]/Total number of plants. **(B)** Photograph of the germinated seed inoculation. The image was taken 7 days after inoculation. Ten biological replicates were used for each strain. **(C)** Variations in cell numbers in the leaf infiltration experiments after 8 days using the colony counting method. Three biological replicates with three technical replicates were evaluated for each strain. **(D)** Photograph of the leaf infiltration experiment. The images were taken 4 and 8 days after inoculation. Different letters above the error bars indicate significant differences according to ANOVA (p<0.05) with Tukey’s HSD test and the error bars indicate the standard errors of means. Five independent experiments were conducted, all of which showed similar patterns.

A Tn5-insertional mutant library generated with the strain KACC17005 was screened to identify genes associated with the virulence of *Ac*. From this library, we selected one mutant strain that did not cause BFB disease symptoms in watermelon and linked this phenotype to a gene encoding a putative 2,3-bisphosphoglycerate-dependent phosphoglycerate mutase (BdpmAc) (Accession No. ATG93870). To confirm the involvement of BdpmAc in the virulence of *Ac*, both germinated seed inoculation and leaf infiltration experiments were conducted. Furthermore, *Ac*(EV), *bdpmAc:Tn*(EV), and *bdpmAc:Tn*(BdpmAc) were constructed to conduct pathogenicity tests. *Ac*(EV) and *bdpmAc:Tn*(EV) were the wild-type strains and *bdpmAc:Tn* was the strain carrying the empty pBBR1-MCS5 vector. *bdpmAc:Tn*(BdpmAc) was the *bdpmAc:Tn* strain that carried the original *bdpmAc* on the pBBR1-MCS5 vector. As illustrated in **Figures 1A, B**, the *bdpmAc:Tn*(EV) strain induced little to no disease symptoms. Conversely, the *Ac*(EV) strain induced severe wilting symptoms, and the *bdpmAc:Tn*(BdpmAc) strain induced some spots and wilting symptoms. Seven days after inoculation, the disease severity index of the *Ac*(EV) strain was 2, whereas that of *bdpmAc:Tn*(EV) was 0.1. The complemented strain recovered to some extent, and the disease severity was 1.5. Similar results were observed in the leaf infiltration experiments compared to the germinated seed inoculation experiments (**Figures 1C, D**). Leaves infiltrated with *Ac*(EV) became dark and exhibited wilting symptoms. However, the leaves infiltrated with *bdpmAc:Tn*(EV) exhibited very mild wilting symptoms. The complemented strain induced similar symptoms in the inoculated leaves to those of the *Ac*(EV) strain. Furthermore, the *bdpmAc:Tn*(EV) strain in the inoculated watermelon leaf produced fewer CFUs than *Ac*(EV) and *bdpmAc:Tn*(BdpmAc) at 0, 2, 4, 6, and 8 days after inoculation. Therefore, our findings indicated that BdpmAc was essential for virulence.

### BdpmAc is required for glycolysis and gluconeogenesis

**Figure 2:**
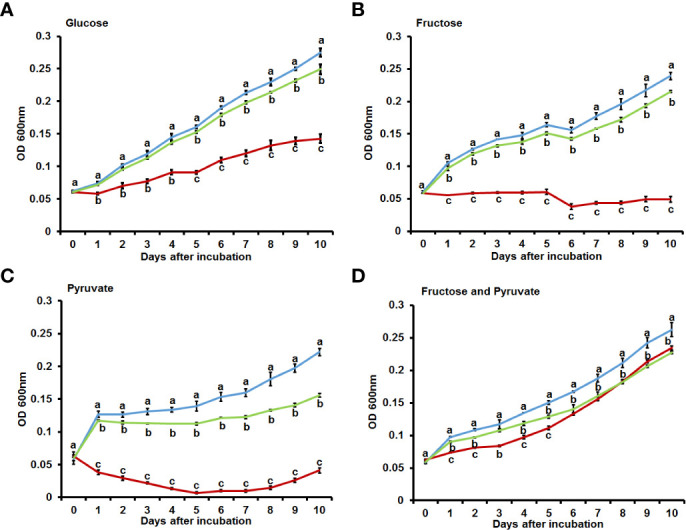
Growth assay in M9 minimal media with diverse carbon sources. Bacterial growth in M9 minimal media with **(A)** glucose (0.4%), **(B)** fructose (0.4%), **(C)** pyruvate (0.4%), and **(D)** fructose (0.2%) and pyruvate (0.2%) as carbon sources. The values at OD_600nm_ were measured for 10 days at 24 h intervals. The error bars from three biological replicates indicate the standard deviation and the different characters above the error bars indicate significant differences according to ANOVA (p<0.05) with Tukey’s HSD test. At least four independent experiments were performed and all experiments displayed similar patterns.

Sequencing analyses confirmed that the above-described phenotypes were attributable to changes in the *bdpmAc* gene that encodes a putative 2,3-bisphosphoglycerate-dependent phosphoglycerate mutase, which is a key enzyme in the glycolysis and gluconeogenesis pathways. Therefore, it can be assumed that BdpmAc may be involved in carbon source metabolism or anabolism, and thus its growth in minimal media with single carbon sources was investigated. To confirm the utilization of carbon sources, the growth of *Ac* strains in M9 minimal media with each glycolysis precursor (glucose, fructose, mannitol, sucrose) and gluconeogenic precursor (pyruvate) was monitored. Before conducting the experiments in the M9 medium, growth curves were measured in rich media (TSB). *Ac*(EV) reached the exponential growth phase in TSB after 12 hours of incubation and later reached the stationary stage (around OD_600nm_ 2) at 72 hours. The complemented strain showed a similar growth curve. The growth of *bdpmAc:Tn*(EV) was slightly decreased compared to *Ac*(EV) and *bdpmAc:Tn*(BdpmAc). At 12 hours, there were no visible differences from other strains. However, its growth rate began to decrease after 12 hours at the stationary stage and the OD_600nm_ was less than 2 (**Supplementary Figure 1A**). Next, the *Ac*(EV)*, bdpmAc:Tn*(EV), and *bdpmAc:Tn*(BdpmAc) strains were examined in the presence of glycolysis precursors (glucose and fructose). In M9 with glucose, the *Ac*(EV) strain grew consistently well. In contrast, the growth of the *bdpmAc:Tn*(EV) strain was markedly reduced compared to *Ac*(EV) and the complemented strain. The growth of the complemented strain recovered to similar levels to those of the *Ac*(EV) strain (**Figure 2A**). In the M9 with fructose, *Ac*(EV) and *bdpmAc:Tn*(BdpmAc) grew well but *bdpmAc:Tn*(EV) did not (**Figure 2B**). In M9 supplemented with pyruvate, the *Ac*(EV) strain grew consistently well. However, the *bdpmAc:Tn*(EV) strain exhibited poor growth rates and often showed a tendency to decline. The growth of the complemented strain recovered to levels similar to those of the *Ac*(EV) strain (**Figure 2C**). As illustrated in **Figures 2B, C**, the *bdpmAc:Tn*(EV) strain did not grow well. Therefore, growth assays were conducted in M9 media containing both fructose and pyruvate. Interestingly, the growth of *bdpmAc:Tn*(EV) recovered to levels similar to those of *Ac*(EV) and the complemented strain (**Figure 2D**). Lastly, we examined the growth of *Ac* strains with mannitol produced *via* hydrogenation of fructose and sucrose, which is composed of glucose and fructose (**Supplementary Figures 1B, D**). None of the examined strains grew well in this medium. These results demonstrate that BdpmAc is essential for both glycolysis and gluconeogenesis in *Ac*.

### Proteomic analysis

**Figure 3:**
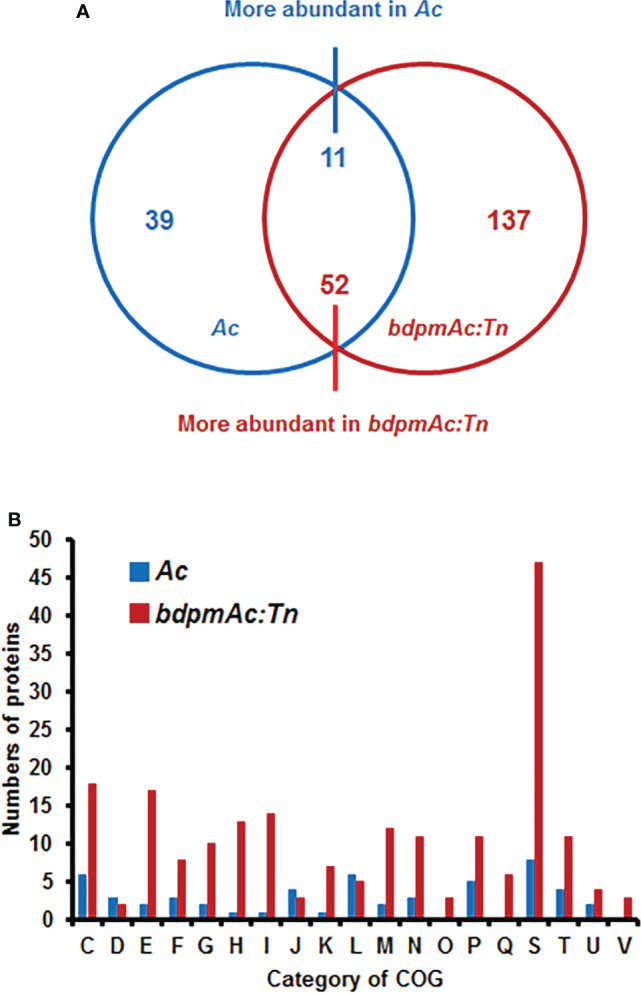
Comparative proteomic analysis between *Ac* and *bdpmAc:Tn*. **(A)** Venn diagram of the differentially (over two-fold change) abundant proteins identified in the comparative proteomic analysis. A total of 39 and 137 proteins were only found in *Ac* and *bdpmAc:Tn*, and 11 and 52 proteins were more abundant in these strains, respectively. **(B)** Categorization of the differentially abundant proteins using the clusters of orthologous groups (COGs). Abbreviations:C, Energy production and conversion; D, Cell cycle control and mitosis; E, Amino acid metabolism and transport; F, Nucleotide metabolism and transport; G, Carbohydrate metabolism and transport; H, Coenzyme metabolism; I, Lipid metabolism; J, Translation; K, Transcription; L, Replication and repair; M, Cell wall/membrane/envelop biogenesis; N, Cell motility; O, Post-translational modification, protein turnover, chaperone functions; P, Inorganic ion transport and metabolism; Q, Secondary structure; S, Function unknown; T, Signal transduction; U, Intracellular trafficking and secretion; V, Defense mechanisms.

To explore the potential cellular mechanisms affected by BdpmAc, a label-free shotgun comparative proteomic analysis followed by COG categorization was conducted using *Ac* and *bdpmAc:Tn*. The numbers of the detected PSMs in the three biological replicates from *Ac* were similar to those of *bdpmAc:Tn* (65,333–65,715) (**Supplementary Table 2**). However, more proteins were identified in *bdpmAc:Tn* than in *Ac* (**Supplementary Table 2**). A total of 929 and 1,045 proteins were detected in all three biological replicates from *Ac* and *bdpmAc:Tn*, respectively (**Supplementary Table 2**). Next, these proteins were subjected to comparative proteomic analysis, which showed that 39 and 137 proteins were only detected in *Ac* and *bdpmAc:Tn*, and 11 and 52 proteins were more abundant (over 2-fold) in these strains, respectively (**Figure 3A**). These differentially abundant proteins were categorized using COG analysis (**Figure 3B**; **Supplementary Table 3** and **4**). BdpmAc was only detected in the wild-type but not in the *bdpmAc:Tn* mutant (**Supplementary Tables 3**, 4), indicating that the comparative proteomic analysis was correctly conducted. The numbers of classified proteins in most categories in *bdpmAc:Tn* were higher than those in *Ac*. Proteins belonging to two categories, J (translation) and L (replication and repair), were more abundant in *Ac*. Interestingly, diverse proteins related to carbohydrate metabolism as well as cell wall/membrane/envelop biogenesis were identified in the comparative proteomic analysis. Additionally, proteins associated with cell movement including pili motility proteins were abundantly detected. The results of our comparative proteomic analysis suggest that BdpmAc is involved in diverse phenotypes in *Ac* including carbohydrate metabolism, biofilm formation, and twitching motility.

### BdpmAc is related to biofilm formation

**Figure 4:**
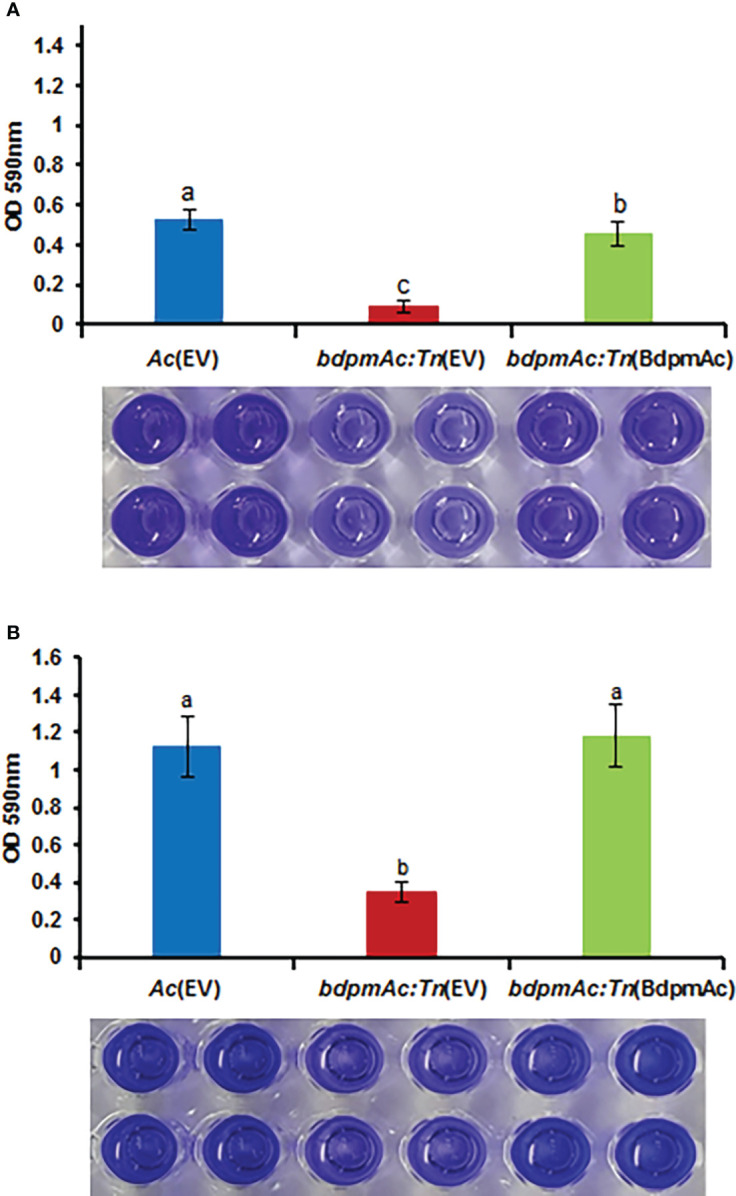
Biofilm formation in *Ac*(EV), *bdpmAc:Tn*(EV), and *bdpmAc:Tn*(BdpmAc). *Ac* strains were incubated in polyvinyl chloride 96-well plates for **(A)** one and **(B)** three days after incubation. For quantification, the formed biofilm was stained using 0.1% crystal violet. The stained biofilm was dissolved in 95% ethanol and measured using a UV spectrophotometer at OD_590nm_. The error bars indicate the standard deviations of twenty biological replicates and different characters above the error bars indicate significant differences according to ANOVA (p<0.05) with Tukey’s HSD test. Eight independent experiments were conducted, all of which exhibited similar patterns.

Our comparative proteomic analyses identified several differentially abundant proteins associated with cell wall/membrane/envelop biogenesis. The bacterial cell wall/membrane is known to be closely related to biofilm formation (Bucher et al., 2015). Additionally, a previous study reported that a phosphoglycerate mutase knockout mutant of *Stenotrophomonas maltophilia* exhibited a decreased biofilm formation capacity (Ramos-Hegazy et al., 2020). Therefore, we next sought to compare the biofilm formation capacity of *Ac*(EV)*, bdpmAc:Tn*(EV), and *bdpmAc:Tn*(BdpmAc) using a 96-well PVC microplate assay. The biofilm formation capacity of the *Ac* strains was evaluated and measured at 1 and 3 days after incubation (DAI). At 1 DAI, biofilm formation significantly differed between *Ac*(EV) and *bdpmAc:Tn*(EV) (**Figure 4A**). Specifically, the biofilm formation ability of *bdpmAc:Tn*(EV) decreased markedly (over 5.9-fold) compared to *Ac*(EV). In contrast, the biofilm formation capacity of *bdpmAc:Tn*(BdpmAc) was similar to that of *Ac*(EV). The results observed at 3 DAI were similar to those observed at 1 DAI (**Figure 4B**). The biofilm formation capacity of *bdpmAc:Tn*(EV) was lower (over 3.2-fold) than that of *Ac*(EV) and *bdpmAc:Tn*(BdpmAc) on day 3. Therefore, our findings demonstrated that BdpmAc is involved in biofilm formation.

### *BdpmAc:Tn*(EV) showed reduced twitching halos

**Figure 5:**
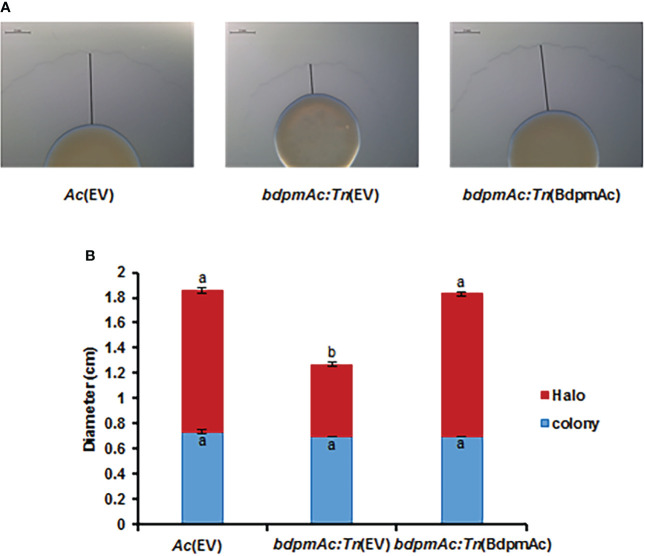
Twitching halo size in *Ac*(EV), *bdpmAc:Tn*(EV), and *bdpmAc:Tn*(BdpmAc). **(A)** Observation of twitching halo size under a stereoscopic microscope. The black lines indicate the length of the twitching halo. Scale bar: 2 mm. **(B)** Measurement of the colony and twitching halo sizes. The blue and red bars indicate the diameter of the colony and twitching halo diameter, respectively. The error bars indicate the standard deviations of three biological replicates. Different characters indicate significant differences according to ANOVA (p<0.05) with Tukey’s HSD test. Eight independent experiments were carried out, all of which exhibited similar patterns.

Many proteins involved in cell motility were detected in the COG categorization. Similar to other gram-negative plant pathogenic bacteria, *Ac* possesses two types of appendages (pili and flagella) as motility organs. However, the Group II strain of *Ac* rarely showed flagella-dependent motility and mostly used pili-dependent motility (Bahar et al., 2010). Therefore, to verify if BdpmAc is related to cell motility, the twitching motility (i.e., pili-dependent motility) assay was conducted. This assay was conducted in TSB with 0.5% agar and the sizes of the bacterial colonies and twitching halos were evaluated. The colony sizes of *Ac*(EV), *bdpmAc:Tn*(EV), and *bdpmAc:Tn*(BdpmAc) were 0.73 cm, 0.7 cm, and 0.7 cm, respectively (**Figure 5**), all of which were deemed to be statistically equal. However, there was a significant difference in the twitching halos between *Ac*(EV) and *bdpmAc:Tn*(EV). The twitching halos of *bdpmAc:Tn*(EV) were smaller (1.26 cm) compared to those of *Ac*(EV) (1.85 cm). In contrast, the twitching halo size of the complemented strain was restored (1.83 cm) and was similar to that of the *Ac*(EV) strain. These results demonstrated that BdpmAc affects the twitching halo size of *Ac*.

### BdpmAc is associated with tolerance to osmotic stress

**Figure 6:**
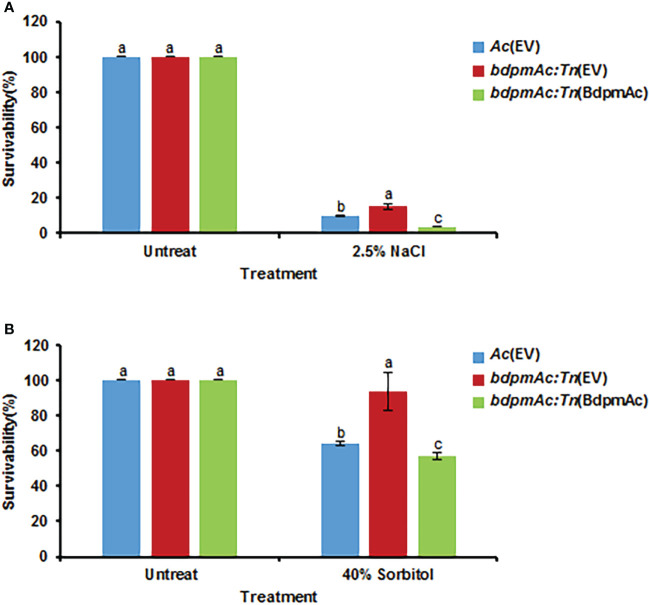
Osmotic stress tolerance. The tolerance of the *Ac* strains to osmotic stresses was evaluated with **(A)** 2.5% NaCl and **(B)** 40% sorbitol in TSB. Bacterial cell survival rates were evaluated *via* colony counting. The survival rate was calculated as the ratio of the viable cell numbers from the stress conditions to those of the untreated samples. The error bars indicate the standard error of the mean from three biological replicates with three technical replicates and different characters above the bars indicate significant differences according to ANOVA (p<0.05) with Tukey’s HSD Test. Seven and five independent experiments were conducted for NaCl and sorbitol, respectively. All experiments showed similar patterns.

As indicated by our COG analyses, proteins categorized in group M (cell wall/membrane/envelope biosynthesis) were more abundant in *bdpmAc:Tn* compared to *Ac.* The bacterial cell envelopes are essential for stabilizing the turgor pressure (Rojas et al., 2018). These findings suggest that *bdpmAc:Tn* is more tolerant to osmotic stress than *Ac.* To test this hypothesis, the three strains were exposed to NaCl and sorbitol. First, the three strains were exposed to 2.5% NaCl in TSB for 20 minutes. *Ac*(EV) exhibited a 9.6% survival rate, whereas *bdpmAc:Tn*(EV) had a 15.3% survival rate (**Figure 6A**). Moreover, the survival rate of the complemented strain was also lower than that of *bdpmAc:Tn*(EV) (3.75%). Next, *Ac* was exposed to 40% sorbitol in TSB for 40 minutes and the results of this assay were consistent with those of the NaCl stress experiments. The *Ac*(EV) strain exhibited a survival rate of 64%, whereas *bdpmAc:Tn*(EV) exhibited a 93.6% survival rate (**Figure 6B**). The survival rate of the complemented strain was also lower than that of *bdpmAc:Tn*(EV) (57%). These data indicate that BdpmAc is associated with in tolerance to osmotic stress.

## Discussion

PGMs are key enzymes in both the glycolysis and gluconeogenesis pathways. These enzymes can be classified into two groups: iPGMs and dPGMs. Moreover, a previous study reported that iPGM was involved in the virulence of certain bacteria (Radin et al., 2019). However, the involvement of dPGMs in bacterial virulence is rarely reported. Using two different pathogenicity assays, our study demonstrated that BdpmAc, which belongs to the dPGM group, is indispensable for virulence in *Ac*. Compared with *Ac*(EV) and *bdpmAc:Tn*(BdpmAc), our findings also demonstrated that *bdpmAc:Tn*(EV) did not grow well in M9 medium with glucose or fructose, which are precursors in the glycolysis pathway. In agreement with our observations, a dPGM knockout mutant in *Burkholderia phymatum* also showed similar growth patterns in a previous study (Chen et al., 2012). Interestingly, *bdpmAc:Tn*(EV) did not grow in M9 with fructose but exhibited moderate to low growth rates in M9 supplemented with glucose. The glycolysis pathway is the first line in carbohydrate metabolism and it produces pyruvate, which is required to generate ATP *via* the TCA cycle (Harris and Harper, 2015). Kersters et al. reported that some bacteria were able to use the Entner-Doudoroff pathway, which is another glucose-catabolizing pathway (Kersters and De Ley, 1968). This pathway can produce pyruvate from glucose without PGMs. Interestingly, a previous studies has reported that this pathway was less efficient than the normal glycolysis pathway in terms of ATP production (Stettner and Segrè, 2013). This means that some bacteria possess an alternative and less efficient pathway to use glucose. Therefore, *bdpmAc:Tn*(EV) was likely able to grow slightly in the presence of glucose without BdpmAc using the aforementioned pathway instead of the normal glycolysis pathway. Phosphogluconate dehydratases and keto-deoxy-phosphogluconate aldolases are known to be required for the Entner-Doudoroff pathway (Conway, 1992). Interestingly, the wild-type *Ac* strain KACC17005 used in this study possesses homologs for the aforementioned enzymes (ATG92716 and ATG92717) (Park et al., 2017). Therefore, it appears that *bdpmAc:Tn*(EV) can use the low-efficiency Entner-Doudoroff pathway, resulting in slow growth rates in the presence of glucose. Unlike the above-described alternative glucose pathway, alternative pathways for fructose metabolism have not been reported in bacteria. Therefore, we could deduce that the *Ac* KACC17005 strain does not possess an alternative pathway for the utilization of fructose because *bdpmAc:Tn*(EV) is not able to grow in the presence of fructose as a sole carbon source.

*Ac*(EV) and *bdpmAc:Tn*(BdpmAc) could grow well in the presence of pyruvate, which is a gluconeogenic and TCA cycle precursor, whereas the growth of *bdpmAc:Tn*(EV) was inhibited. In a previous study, the growth of a dPGM knockout mutant of *B. phymatum* was restored when provided with a mixture of both glycolysis and gluconeogenesis precursors in minimal media (Chen et al., 2012). In agreement with this observation, the growth rates of *bdpmAc:Tn*(EV) were comparable to those of *Ac*(EV) and *bdpmAc:Tn*(BdpmAc). These results indicate that BdpmAc is required for both the glycolysis and gluconeogenesis pathways. Additionally, neither of the strains grew well in the presence of mannitol and sucrose, which are high molecular weight compounds. These findings indicate that *Ac* cannot use high molecular weight compounds for glycolysis and therefore could not utilize either of the aforementioned compounds as a carbon source. Previous studies have also reported that *Ac* cannot use mannitol and sucrose as sole carbon sources (Melo et al., 2014). Taken together, our findings suggested that *Ac* possesses an alternative pathway for glucose metabolism, and BdpmAc is a key enzyme for the glycolysis and gluconeogenesis pathways in *Ac*. The growth of *bdpmAc:Tn*(EV) in rich media (TSB) was slightly attenuated. Furthermore, in the comparative proteomic analysis, the proteins belonging to group D (cell cycle control/cell division/chromosome partitioning) in *Ac* were more abundant than those in *bdpmAc:Tn*. These findings indicate that BdpmAc may affect bacterial multiplication and reproduction. However, the virulence of the mutant was dramatically reduced in both the germinated seed assay and the leaf infiltration assay. Therefore, it can be speculated that there are other mechanisms related to BdpmAc functions that may contribute to the virulence of *Ac*.

Felgner et al. demonstrated that AroA was required for amino acid synthesis and other cellular processes including virulence in *Salmonella enterica* serovar Typhimurium (Felgner et al., 2016). Similarly, another study reported that a putative bifunctional chorismate mutase/prephenate dehydratase, CmpAc, is involved in virulence in *Ac* and other mechanisms such as the biosynthesis of phenylalanine, twitching, biofilm formation, motility, and tolerance to stresses (Kim et al., 2020). These studies demonstrate that one protein is associated with several distinct cellular mechanisms, meaning that it displays pleiotropic effects. Likewise, our comparative proteomic analyses and phenotypic assays also demonstrated that BdpmAc possesses pleiotropic effects in *Ac*.

Our proteomic analyses revealed that the abundance of diverse proteins categorized in group N (cell motility) was altered by BdpmAc. Similar to other bacteria, *Ac* also exhibits flagella- and pili-dependent motility. However, *Ac* strains categorized in Group II exhibited only pili-dependent motility (i.e., twitching motility) but not flagella-dependent motility in laboratory conditions (Bahar et al., 2010). Because the wild-type KACC17005 strain used in this study belongs to Group II (Kim et al., 2020), we characterized twitching motility in rich medium and found that *bdpmAc:Tn*(EV) exhibited a reduction in twitching motility. This reduction in twitching motility might explain the low virulence of the mutant. Other studies also support this speculation. For instance, the loss of genes associated with twitching motility decreased the virulence of *Ac* in two previous studies (Bahar et al., 2009; Rosenberg et al., 2018). Twitch motility is known to be involved in biofilm formation, which is an important determinant of virulence in prokaryotic organisms (Davey and O’toole, 2000). A previous study demonstrated that the *pgm* mutant of *Stenotrophomonas maltophilia* displayed an impaired biofilm formation capacity (Ramos-Hegazy et al., 2020). In agreement with the aforementioned report, the biofilm formation ability of *bdpmAc:Tn*(EV) was dramatically reduced compared to the wild-type and the complemented strains. Taken together, our findings suggest that BdpmAc affects twitching motility and biofilm formation, which may in turn affect the virulence of *Ac*. Interestingly, many proteins classified in Group M (cell wall/membrane/envelope biosynthesis) were identified in the comparative proteomic analysis. Additionally, *bdpmAc:Tn*(EV) exhibited a higher tolerance against NaCl- and sorbitol-induced osmotic stress, thus demonstrating that BdpmAc is involved in cell wall/membrane functions. Virulence and tolerance to osmotic stress are known to be positively correlated in gram-negative bacteria (Park et al., 2019; Kim et al., 2020). However, the tolerance of *bdpmAc:Tn*(EV) against osmotic stress was accompanied by a decrease in virulence in our study. Although the mechanisms behind this seemingly conflicting phenomenon remain unclear, we can speculate that BdpmAc is involved in other cellular processes and mechanisms in addition to modulating virulence.

Using comparative proteomic analysis and phenotypic assays, we demonstrated that a putative 2,3-bisphosphoglycerate-dependent phosphoglycerate mutase, BdpmAc, is indispensable for virulence, glycolysis, and gluconeogenesis in *Ac*. Furthermore, BdpmAc is also related to biofilm formation and twitching motility. We also showed that BdpmAc may possess pleiotropic effects in *Ac*. Particularly, this study suggests that BdpmAc, which is involved in carbohydrate metabolism, may also be an important virulence factor. Therefore, our findings provide novel insights into the functions of a phosphoglycerate-dependent phosphoglycerate mutase, which could be a potential target to develop anti-virulence agents to control BFB.

## Data availability statement

The mass spectrometry proteomics data have been deposited to the ProteomeXchange Consortium via the PRIDE (Perez-Riverol et al., 2022) partner repository with the dataset identifier PXD035156.

## Author contributions

S-WH conceived the study. S-WH and JCL designed the experiments. JCL, JWL, YC, and JC conducted the experiments. JCL and S-WH analyzed the data and prepared the manuscript. All authors contributed to the article and approved the submitted version.

## Funding

This work was supported by the National Research Foundation of Korea (NRF) grant funded by the Korean government (MSIT) (No. NRF-2020R1A2C1013040), Republic of Korea. This research was also supported by the Chung-Ang University Graduate Research Scholarship in 2022 to JWL.

## Conflict of interest

The authors declare that the research was conducted in the absence of any commercial or financial relationships that could be construed as a potential conflict of interest.

## Publisher’s note

All claims expressed in this article are solely those of the authors and do not necessarily represent those of their affiliated organizations, or those of the publisher, the editors and the reviewers. Any product that may be evaluated in this article, or claim that may be made by its manufacturer, is not guaranteed or endorsed by the publisher.

## Supplementary material

Supplementary Figure 1 Growth assay in TSB and M9 minimal media with mannitol and sucrose. Bacterial growth in **(A)** TSB and M9 minimal media with **(B)** mannitol (0.4%) and **(C)** sucrose (0.4%). The values at OD_600nm_ were evaluated for five and ten days in TSB and minimal media, respectively. The error bars from three biological replicates indicate the standard deviation and different characters above the error bars indicate significant differences according to ANOVA (p<0.05) with Tukey’s HSD test. At least four independent experiments were performed, all of which exhibited similar patterns.
