# Supplementary figures and images for: A putative 2,3-bisphosphoglycerate-dependent phosphoglycerate mutase is involved in the virulence, carbohydrate metabolism, biofilm formation, twitching halo, and osmotic tolerance in Acidovorax citrulli
Source: Front Plant Sci. 2022 Nov 9;13:1039420. doi: 10.3389/fpls.2022.1039420 (PMC9681784; doi:10.3389/fpls.2022.1039420)

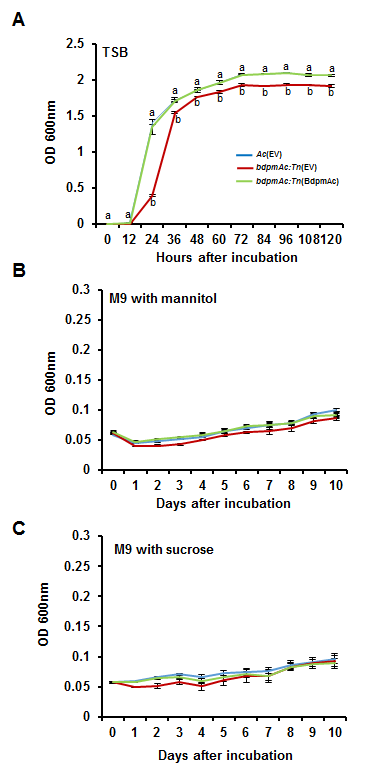

Supplement: Supplementary Figure 1 — Growth assay in TSB and M9 minimal media with mannitol and sucrose. Bacterial growth in (A) TSB and M9 minimal media with (B) mannitol (0.4%) and (C) sucrose (0.4%). The values at OD600nm were evaluated for five and ten days in TSB and minimal media, respectively. The error bars from three biological replicates indicate the standard deviation and different characters above the error bars indicate significant differences according to ANOVA (p<0.05) with Tukey’s HSD test. At least four independent experiments were performed, all of which exhibited similar patterns. [file Image_1.tif]
