# Supplementary material for: A putative 2,3-bisphosphoglycerate-dependent phosphoglycerate mutase is involved in the virulence, carbohydrate metabolism, biofilm formation, twitching halo, and osmotic tolerance in Acidovorax citrulli
Source: Front Plant Sci. 2022 Nov 9;13:1039420. doi: 10.3389/fpls.2022.1039420 (PMC9681784; doi:10.3389/fpls.2022.1039420)
Supplement: Supplementary file 2 [file Table_1.docx]

| **Supplementary Table 1.** Bacterial strains and plasmids used in this study. | | |
| --- | --- | --- |
| Strains or plasmids | Characteristic(s) | Source or reference |
| *Escherichia coli* |  |  |
| EC100D | Strain used for rescuing EZ:Tn transposon inserted plasmid | Epicentre |
| DH5α | Strain used for cloning | Promega |
|  |  |  |
| *Acidovorax citurlli* |  |  |
| KACC17005 | Wild type, complete whole genome sequence, Rif^r^ | (Park et al. 2017) |
| *Ac*(EV) | Wild type carrying the pBBR1-MCS5, Rif^r^, Gm^r^ | This study |
| *bdpmAc:Tn* | Knock out mutant, EZ:Tn transposon inserted in *bdpmAc*, Rif^r^, Kan^r^ | This study |
| *bdpmAc:Tn*(EV) | *bdpmAc:Tn* carrying the pBBR1-MCS5, Rif^r^, Kan^r^, Gm^r^ | This study |
| *bdpmAc:Tn*(BdpmAc) | Complemented strain, *bdpmAc:Tn* carrying the MCS5-*bdpmAc*, Rif^r^, Kan^r^, Gm^r^ | This study |
|  |  |  |
| Plasmid |  |  |
| pGem-T easy | T-A cloning vector, Amp^r^ | Promega |
| pGem-*bdpmAc* | pGem-T easy vector ligating 773bp of *bdpmAc*, Amp^r^ | This study |
| pBBR1-MCS5 | Broad-host-range vector, LacZ promoter, Gm^r^ | (Kovach et al. 1995) |
| pMCS5-*bdpmAc* | pBBR1-MCS5 carrying the *bdpmAc* from pGem- *bdpmAc* plasmid, Gm^r^ | This study |
| Rif^r^, Kan^r^, Gm^r^ and Amp^r^ represent resistance to Rifampicin, Kanamycin, Gentamycin and Ampicillin, respectively | | |
