# Supplementary material for: A putative 2,3-bisphosphoglycerate-dependent phosphoglycerate mutase is involved in the virulence, carbohydrate metabolism, biofilm formation, twitching halo, and osmotic tolerance in Acidovorax citrulli
Source: Front Plant Sci. 2022 Nov 9;13:1039420. doi: 10.3389/fpls.2022.1039420 (PMC9681784; doi:10.3389/fpls.2022.1039420)
Supplement: Supplementary file 3 [file Table_2.docx]

**Supplementary Table 2.** Proteins and peptide spectral matches (PSMs) identified in liquid chromatography with tandem mass spectrometry.

| Strain | 1st | | 2nd | | | | 3rd | | shared proteins in 3 biological replicates |
| --- | --- | --- | --- | --- | --- | --- | --- | --- | --- |
|  | Protein | PSM | | Protein | PSM | Protein | | PSM |  |
| *Ac* | 975 | 65,333 | | 984 | 65,344 | 985 | | 65,295 | 929 |
| *bdpmAc:Tn* | 1,091 | 65,715 | | 1,133 | 65,592 | 1,123 | | 65,586 | 1,045 |
